# Supplementary figures and images for: Applications of the indole-alkaloid gramine modulate the assembly of individual members of the barley rhizosphere microbiota
Source: PeerJ. 2021 Nov 22;9:e12498. doi: 10.7717/peerj.12498 (PMC8614190; doi:10.7717/peerj.12498)

# Non-linearized isotherm adsorption models – Gramine in Quarryfield soil

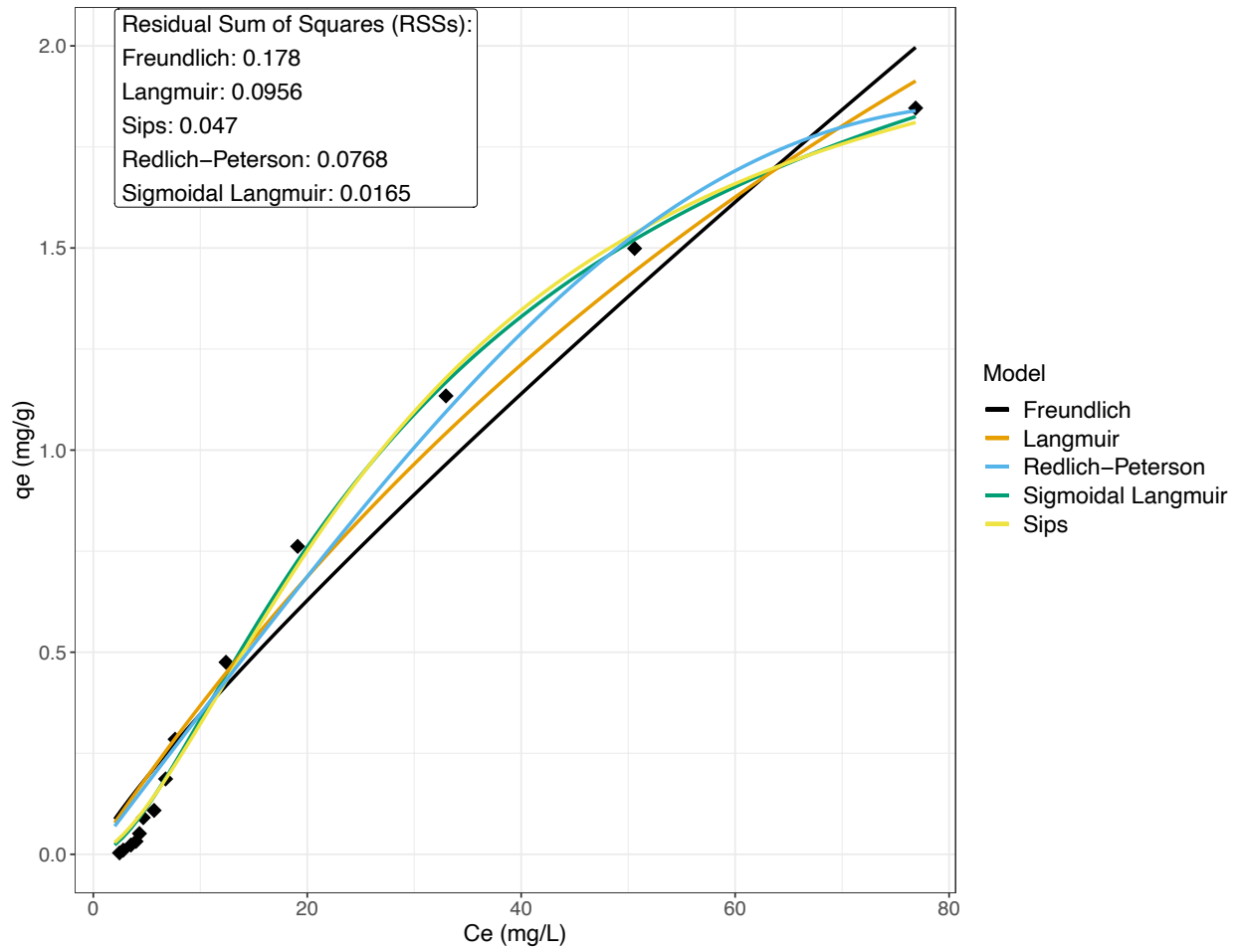

Supplement: Supplemental Information 1 — Adsorption isotherms of gramine in Quarryfield soil were fitted applying several nonlinear models: the two-parameter Langmuir and Freundlich isotherms and the three-parameter Sigmoidal Langmuir, Redlich-Peterson and Sips isotherms, along with their corresponding Residual Sum of Squares (RSSs) indicating the fitness of the model to the data. Ce = gramine concentration at equilibrium, qe = gramine concentration adsorbed on the soil. [file peerj-09-12498-s001.pdf]

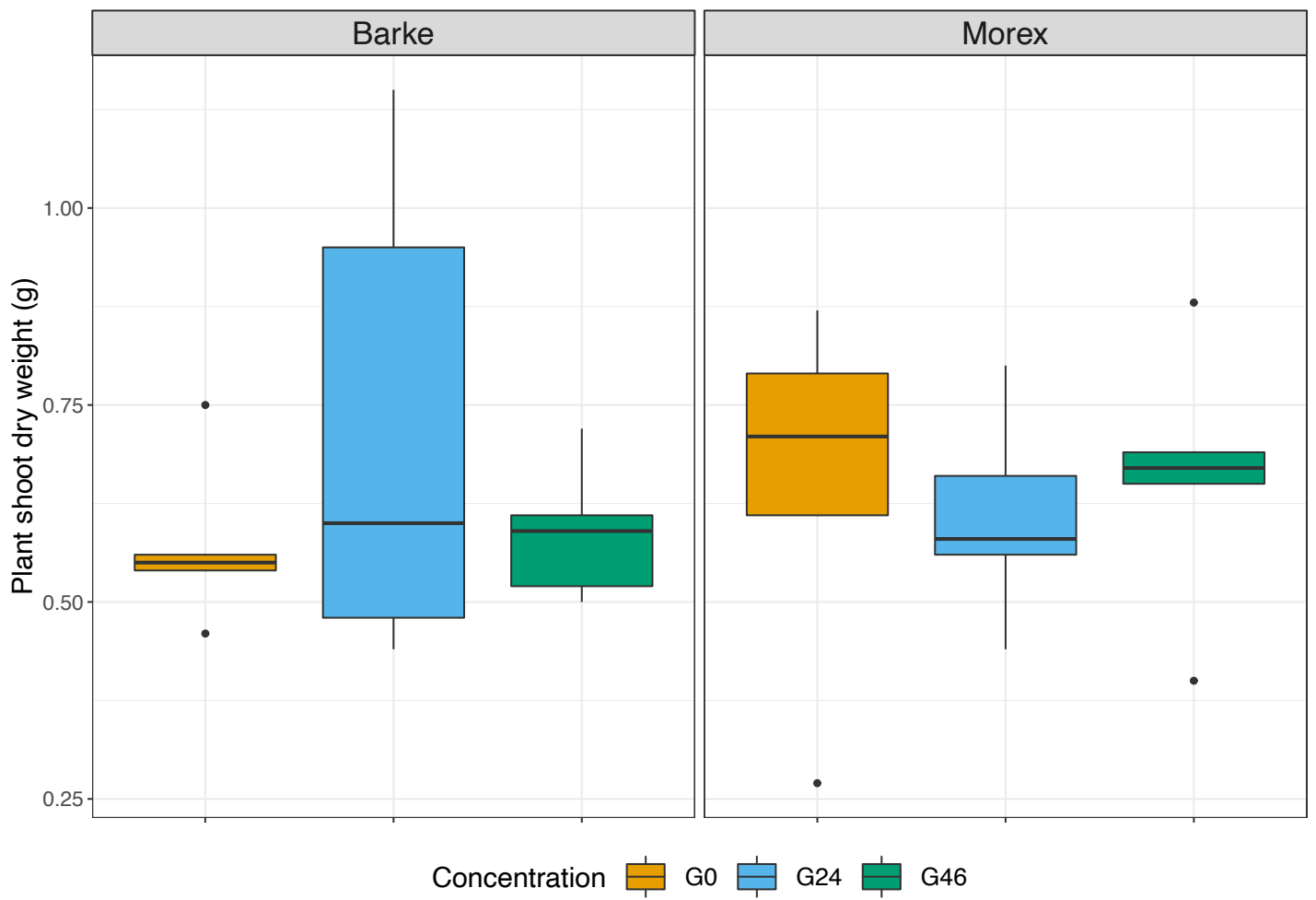

Supplement: Supplemental Information 2 — Stem dry weight of Barke and Morex barley plants subjected to different gramine concentrations at the time of sampling. No significant differences using ANOVA followed by Tukey HSD test, p value > 0.05 [file peerj-09-12498-s002.pdf]

PCoA 16S data, Bray distance

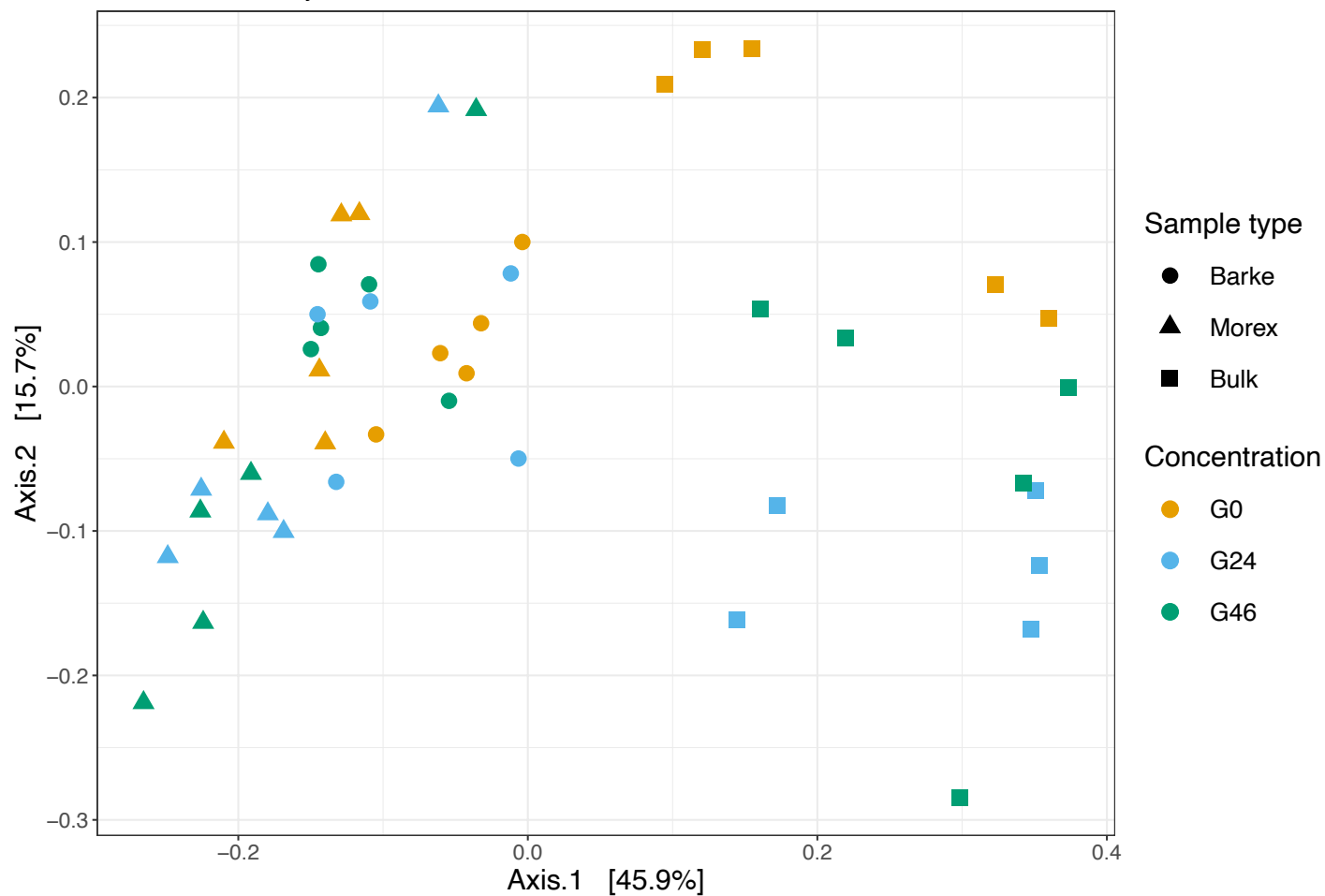

Supplement: Supplemental Information 3 — Individual shapes depict individual samples, color-coded according the gramine treatment imposed on them. The ordination was constrained for genotype and gramine concentrations. [file peerj-09-12498-s003.pdf]

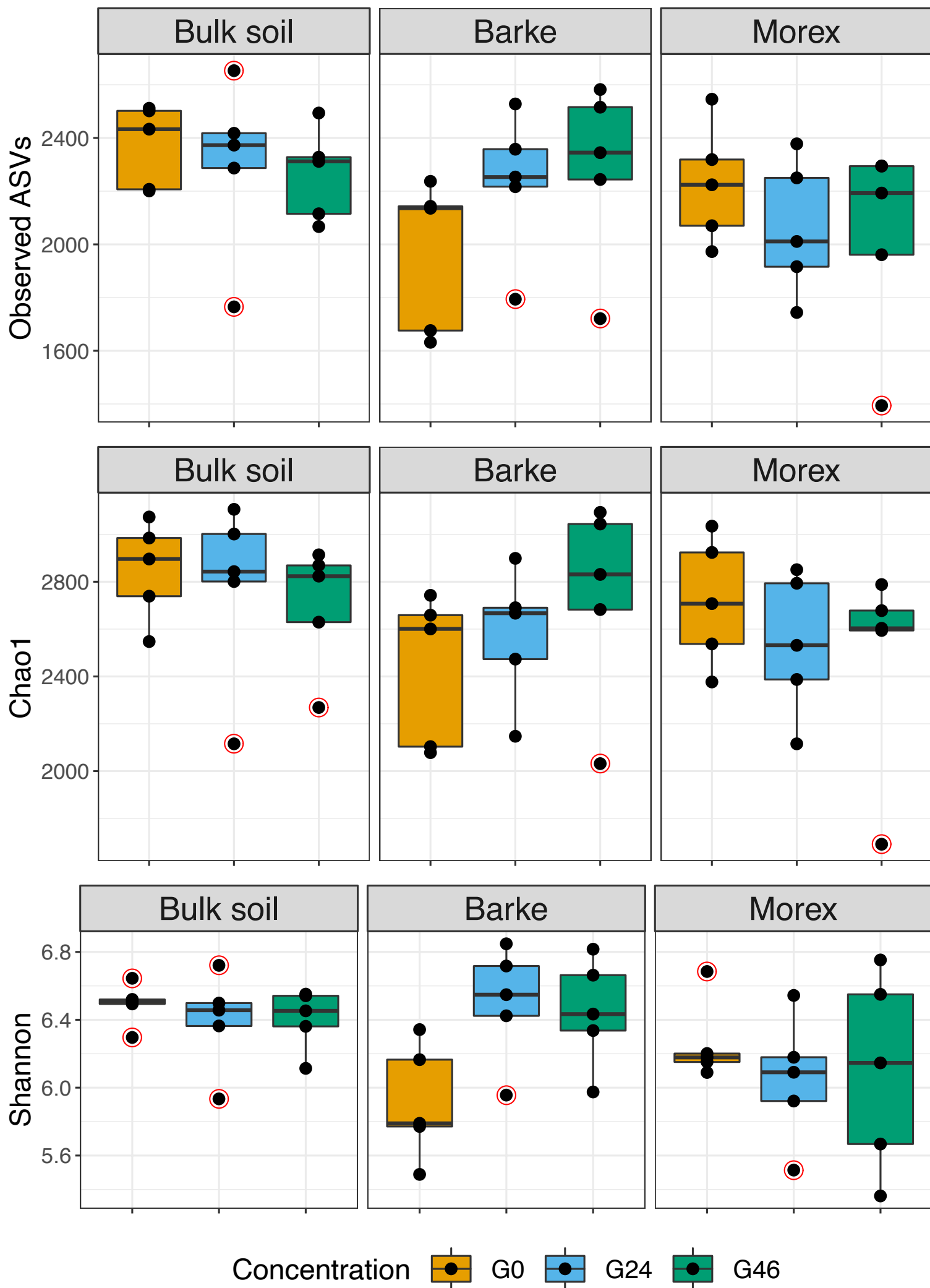

Supplement: Supplemental Information 4 — Alpha diversity Observed ASVs, Chao1 and Shannon indexes, of Bulk soil, Barke and Morex, at three different gramine concentrations. Individual dots depict individual biological replicates; no significant differences observed. [file peerj-09-12498-s004.pdf]

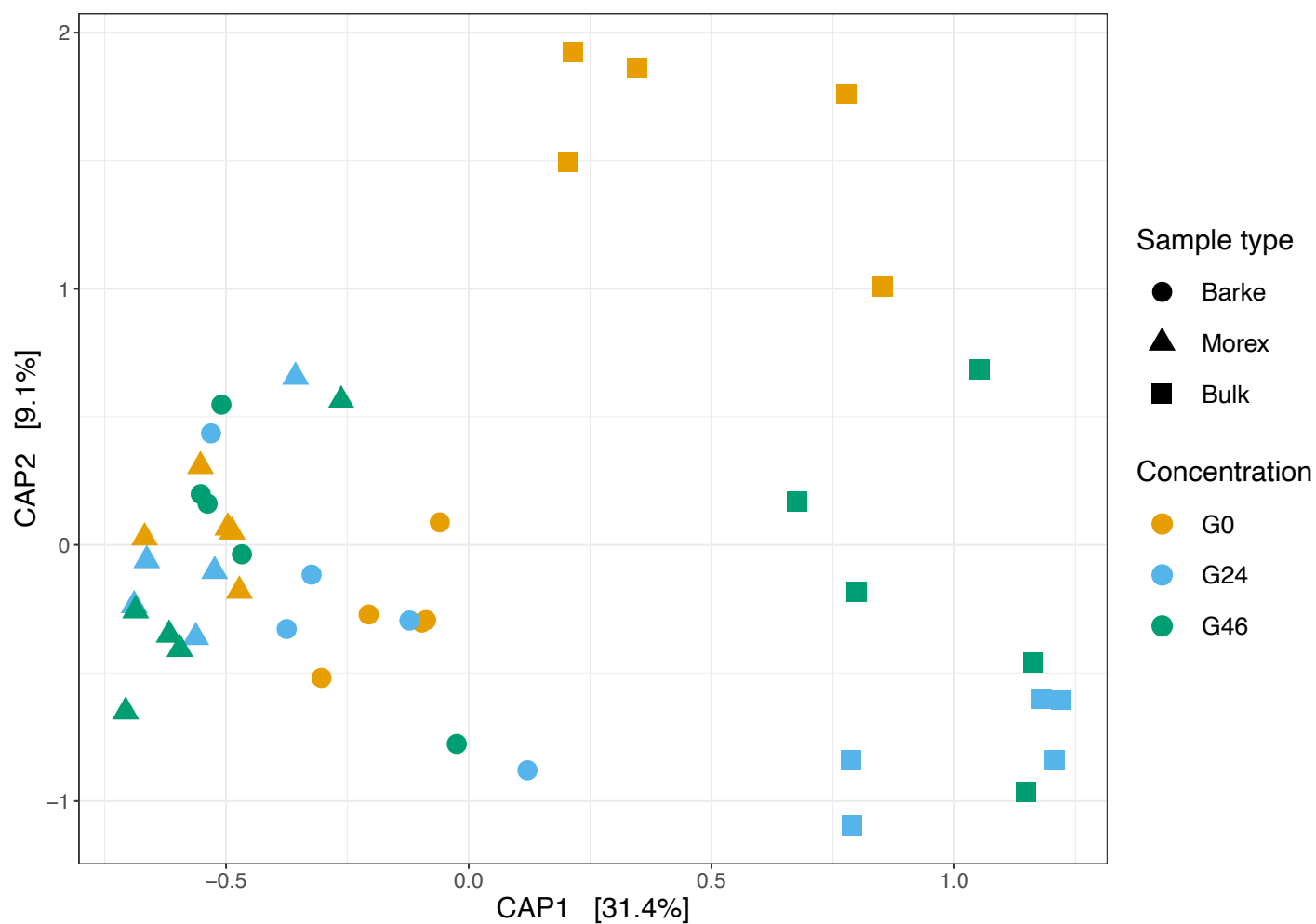

Supplement: Supplemental Information 5 — Individual shapes depict individual samples, color-coded according the gramine treatment imposed on them. The ordination was constrained for genotype and gramine concentrations. [file peerj-09-12498-s005.pdf]
